# Micromanipulation and Automatic Data Analysis to Determine the Mechanical Strength of Microparticles

**DOI:** 10.3390/mi13050751

**Published:** 2022-05-10

**Authors:** Zhihua Zhang, Yanping He, Zhibing Zhang

**Affiliations:** 1 School of Chemical Engineering, University of Birmingham, Birmingham B15 2TT, UK; zxz836@student.bham.ac.uk; 2 Changzhou Institute of Advanced Manufacturing Technology, Changzhou 213164, China; 3 School of Chemical Engineering, Kunming University of Science and Technology, Chenggong Campus, Kunming 650504, China; grace.he1985@hotmail.com

**Keywords:** micromanipulation, automatic data analysis, mechanical strength, microparticles, algorithms

## Abstract

Microparticles are widely used in many industrial sectors. A micromanipulation technique has been widely used to quantify the mechanical properties of individual microparticles, which is crucial to the optimization of their functionality and performance in end-use applications. The principle of this technique is to compress single particles between two parallel surfaces, and the force versus displacement data are obtained simultaneously. Previously, analysis of the experimental data had to be done manually to calculate the rupture strength parameters of each individual particle, which is time-consuming. The aim of this study is to develop a software package that enables automatic analysis of the rupture strength parameters from the experimental data to enhance the capability of the micromanipulation technique. Three algorithms based on the combination of the “three-sigma rule”, a moving window, and the Hertz model were developed to locate the starting point where onset of compression occurs, and one algorithm based on the maximum deceleration was developed to identify the rupture point where a single particle is ruptured. Fifty microcapsules each with a liquid core and fifty porous polystyrene (PS) microspheres were tested in order to produce statistically representative results of each sample, and the experimental data were analysed using the developed software package. It is found that the results obtained from the combination of the “3*σ* + window” algorithm or the “3*σ* + window + Hertz” algorithm with the “maximum-deceleration” algorithm do not show any significant difference from the manual results. The data analysis time for each sample has been shortened from 2 to 3 h manually to within 20 min automatically.

## 1. Introduction

Microparticles are widely used in many functional products in the industry [1]. Measuring their mechanical strength is essential to optimizing their performance during manufacturing, processing, and end-use applications [2]. For example, microcapsules with self-sensing agents used to produce smart structural composites [3,4,5] should be mechanically strong enough to survive different engineering processing steps leading to their incorporation into the composites but weak enough to break after mechanical damage is occurring to the composites so that the need for repair can be indicated quickly. Understanding the mechanical strength of the self-sensing microcapsules plays a crucial role in ensuring the functionalities of the composites. Furthermore, characterizing the mechanical strength of other microparticles, e.g., perfume microcapsules for fabric softeners and detergents [6], and microspheres for chromatography media for bio-separation [7], can also provide essential technical data for new product development and production as well as help to optimize their functionality and performance in end-use applications.

Experimental techniques to determine the mechanical strength of microparticles can be classified as ensemble test methods and single-particle test methods [1,2,8]. The former methods are relatively quick as a group of particles are tested simultaneously, but only the average mechanical strength values can be obtained. The latter methods test particles one by one; thus, their mechanical strength distribution can be obtained, which is crucial in many applications to optimize their functionality and performance. Several techniques have been developed to determine the mechanical strength of single particles, including optical/magnetic tweezers [9], pressure probe [10,11], micropipette aspiration [12,13], atomic force microscopy (AFM) [14,15], nanoindentation [16,17], and micromanipulation based on diametrical compression [18]. The main difference among them lies in the different deformations, which can be generated, and magnitudes of forces, which can be measured. For example, the typical force measured by micromanipulation is from μN to N, while the force by AFM is from pN to μN [2]. Consequently, micromanipulation can provide the rupture strength parameters by compressing particles to break, while it is difficult for other techniques to do so [1].

The micromanipulation technique was firstly developed to test the rupture strength of single mammalian cells [18] and since then has been modified to test the mechanical and surface properties, including the elasticity, plasticity, viscoelasticity, adhesion, and cohesion of a variety of biological and non-biological micro-materials [1], e.g., microcapsules [19,20,21], microspheres [7,22,23], microbeads [24], pollen grains [25], yeast cells [26,27], chondrocytes and chondrons [28,29], biofilms [30,31], fouling deposits [32,33,34,35], and microneedles [36]. It has provided essential technical data to a number of global companies to assist their micro-product development and also played a very important role in academic research to develop new applications of various micro-materials [1,37].

**Figure 1:**
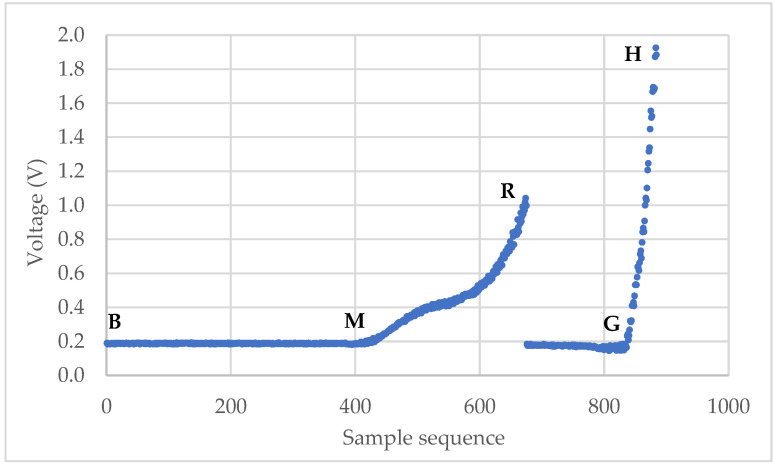
Typical curve of voltage versus sampling sequence from compression of single particles.

The micromanipulation technique involves sample preparation, compression of single particles, and data analysis to obtain the mechanical properties of microparticles. The raw data from a micromanipulation test are a series of voltage data as shown in Figure 1. The main task of the data analysis is to identify the starting point M, where the onset of loading occurs, and rupture point R, where the tested particle ruptured, from which the rupture strength parameters and force-displacement data can be obtained. Unlike some commercial or open-source software packages to analyse the force-displacement data from AFM experiments [38,39], it was carried out manually by interacting with the raw data and template spreadsheets to obtain the results from the micromanipulation experiments, which is quite laborious and time-consuming.

The software packages for AFM are not easy to be adapted to process the data from the micromanipulation technique because of the differences in data formats, mechanical property parameters to be obtained, and specific mathematical model formulas required to be used. However, similar to the starting point M in the micromanipulation tests, the contact point (CP) is also crucial to analysing the force-displacement data from AFM experiments. Several algorithms have been developed to locate the CP of the force-displacement data obtained from AFM experiments. A simple algorithm with a threshold (typically 0.1%) was used to estimate the CP from the approach curve above the baseline [40]. However, this threshold needs to be modified according to the baseline value and noise level manually, which is not suitable for automatic data analysis. A local regression-based algorithm was then introduced to determine the CP by slope changes [41]. Three parameters, including the number of data points for regression, and two thresholds need to be properly set to locate the CP. An algorithm was developed to estimate the CP by fitting the data in a liner elastic region to a Hertz-like model for the nano indentation data [42]. The algorithm worked well but requires new sets of parameters for other materials of different mechanical behaviours. Moreover, in AFM force data analysis, usually a force map, e.g., 64 × 64 force curves, are obtained for a single particle to yield a spatial distribution of the mechanical strength parameters. These algorithms above are aimed to locate the CPs for the force curves for a single particle so that the parameters set for the algorithms may not need to be adjusted frequently for every force curve. In contrast, from micromanipulation measurements, a single voltage (force) curve is obtained for a single particle, and usually, the particles in a sample have different sizes and mechanical strength values; therefore, the parameters set may need to be modified frequently for each dataset to ensure the above algorithms can work properly for every tested single particle in a sample. Consequently, the algorithms used in AFM data analysis cannot be applied directly to automatic analysis of the micromanipulation data.

The aim of this study is to develop a software package to analyse the experimental data obtained from using the micromanipulation technique to automatically obtain the mechanical strength parameters of microparticles to simplify the procedure, save time and labour, and enhance the capability of the micromanipulation technique.

In this paper, three algorithms are presented to identify the starting point M, and an algorithm is introduced to locate the rupture point R from the raw voltage data of micromanipulation. Two samples of microparticles, i.e., the microcapsules for self-sensing and the porous PS microspheres with various potential applications, have been tested using the micromanipulation technique, and the experimental data analysed using the developed software package are compared to the manual results to validate the algorithms developed.

## 2. Materials and Methods

### 2.1. Microparticles for Micromanipulation

#### 2.1.1. Microcapsules for Self-Sensing

The microcapsules for self-sensing were a very robust type of double-walled microcapsules made by interfacial polymerization. The detailed fabrication methods are described in [5]. The outer and inner shells were made from urea formaldehyde (PUF) and polyurethane (PU), respectively. The core is oil with a fluorophore substance.

#### 2.1.2. Porous Polystyrene Microspheres

The porous polystyrene (PS) microspheres with various potential applications were fabricated via a novel solvent evaporation methodology based on foaming transfer. The detailed fabrication process is reported in [43]. Specifically, the porous PS microspheres obtained by introducing 20 wt% ethanol concentration to the continuous phrase were used in this paper.

### 2.2. Micromanipulation of the Microparticles

#### 2.2.1. Micromanipulation Rig

**Figure 2:**
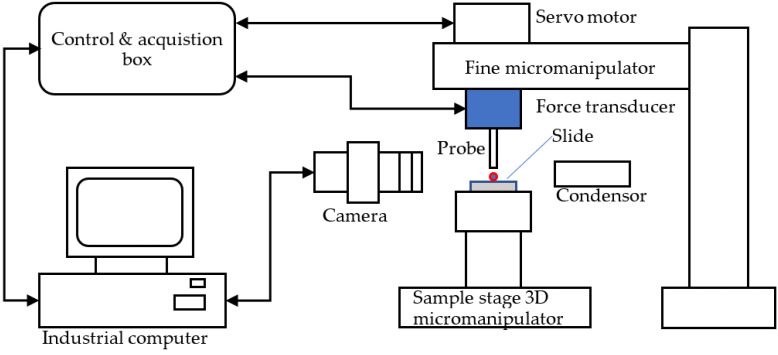
Schematic diagram of the micromanipulation rig.

The principle of the micromanipulation technique is to compress single particles to different deformations or rupture between two parallel surfaces, and the force versus displacement data are obtained simultaneously. The schematic diagram of the micromanipulation rig used in this work is illustrated in Figure 2, which is also reported elsewhere [19,20,21,22]. Single microparticles are placed on the glass slide, which is fixed on the sample stage of a three-dimensional micromanipulator, and then compressed by the output probe (with flat end) of the force transducer that is mounted to the one-dimensional fine micromanipulator. The corresponding compression force is acquired by a data acquisition device (USB-201-OEM, Measurement Computing Corporation, Norton, MA, USA) in the control and acquisition box and the data is saved in the computer for post processing. The fine micromanipulator is driven by a servo motor. The power of the servo motor is 24 V DC. Before compression, single microparticles are moved to just below the force probe by operating the sample-stage micromanipulator. Using the sideview camera, the video images of the compression procedure can be displayed by the industrial computer monitor and saved in the computer. The force transducer can be changed according to the mechanical strength scale of the microparticles to be measured.

#### 2.2.2. Micromanipulation of the Microcapsules for Self-Sensing

Dry microcapsules were placed onto a glass slide, and single microcapsules were compressed to rupture using the micromanipulation rig at a compression speed of 2.0 μm/s. The sampling time was 0.01887 s, and the force transducer model was GS0-10 (Transducer Techniques, LLC, Temecula, CA, USA) with a pre-calibrated sensitivity of 8.674 mN/V. In total, 50 microcapsules were tested at ambient temperature of 26 ± 2 °C.

#### 2.2.3. Micromanipulation of the Porous PS Microspheres

The micromanipulation procedure of the porous PS microspheres was the same as the measurement of the self-sensing microcapsules. The transducer used was GS0-10 with a pre-calibrated sensitivity of 7.423 mN/V. In total, 50 PS microspheres were tested under ambient temperature of 16 ± 2 °C.

**Figure 3:**
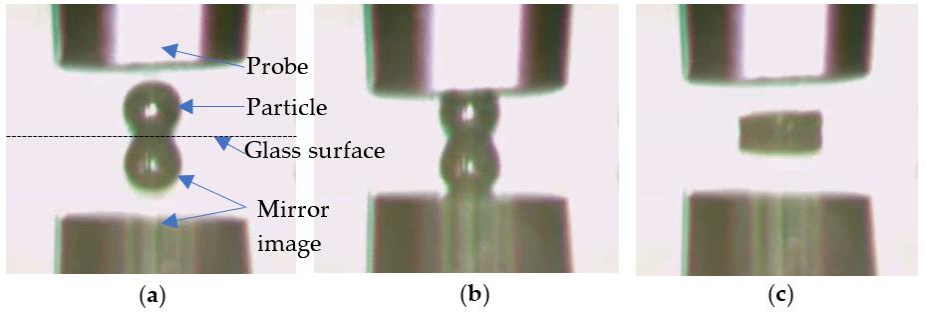
A porous PS microsphere before (**a**), during (**b**), and after (**c**) compression.

Figure 3 illustrates the procedure to compress a porous PS microsphere between the two parallel surfaces, i.e., the probe end and the glass surface. The diameter of the transducer probe was around 50 µm, and the diameter of the particle was 16.3 µm.

### 2.3. Rutpure Strength of Microparticles

The raw data from a micromanipulation test is a series of voltage versus sample sequence data $\left(V_{1},\;V_{2},\;\ldots ,\;V_{n}\right),$ where *n* is the number of the voltage data points. A typical curve is shown in Figure 1. At the beginning, the voltage remains stable along the baseline as the probe moves in the air due to the initial gap between the probe and the microparticle. Then, it starts to increase at M when the probe begins to touch the particle. The voltage keeps rising until R and drops suddenly when the particle is ruptured. After that, the voltage rises again from G as the probe compresses the debris of the particle on the hard bottom surface and stops at H when the voltage limit is reached, or the movement is stopped manually. Point M is named as the starting point and R as the rupture point. The line segment BM is termed as “baseline”. The main task of the data analysis is to identify the starting point M and rupture point R from which the rupture strength parameters, including displacement at rupture $\delta_{r}$, rupture force $F_{r}$, fractional deformation at rupture $\varepsilon_{r}$, nominal rupture stress $\sigma_{r}$, nominal rupture tension $T_{r}$, and toughness $T_{C}$, can be calculated using the following equations

(1) $$F_{r}=s\left(V_{r}-V_{B}\right)$$

(2) $$\delta_{r}=vT_{s}\left(r-m\right)-cF_{r}$$

(3) $$\varepsilon_{r}=\frac{\delta_{r}}{D},$$

(4) $$\sigma_{r}=\frac{4F_{r}}{\pi D^{2}},$$

(5) $$T_{r}=\frac{F_{r}}{D},$$

and

(6) $$T_{C}=\int_{0}^{\varepsilon_{r}}\sigma d\varepsilon ,$$

where m is the starting point index, r is the rupture point index, $V_{r}$ is the voltage corresponding to rupture, $V_{B}$ is the average voltage of the baseline, v is the compression speed, $T_{s}$ is the sampling time, s is the sensitivity of the force transducer, c is the compliance of the force transducer, D is the initial diameter of the single microparticle, σ is the nominal stress, and ε is the fractional deformation.

The force-displacement data can be obtained using the following two equations:

(7) $$F_{i}=s\left(V_{i+m}-V_{B}\right),$$

and

(8) $$\delta_{i}=ivT_{s}-cF,$$

where i (1≤i≤n−m) is the index, F is the compression force, and δ is the displacement. Then, the nominal stress and fractional deformation can be calculated using

(9) $$\sigma_{i}=\frac{4F_{i}}{\pi D^{2}},$$

and

(10) $$\varepsilon_{i}=\frac{\delta_{i}}{D},$$

In practice, the microparticle toughness in Equation (6) can be determined using the trapezoidal numerical integration as Equation (11).

(11) $$T_{C}=\frac{1}{2}\sum_{i=1}^{r}\left(\sigma_{i}+\sigma_{i+1}\right)\left(\varepsilon_{i+1}-\varepsilon_{i}\right),$$

### 2.4. Algorithms to Locate the Starting Point

#### 2.4.1. “3*σ*” Algorithm

**Figure 4:**
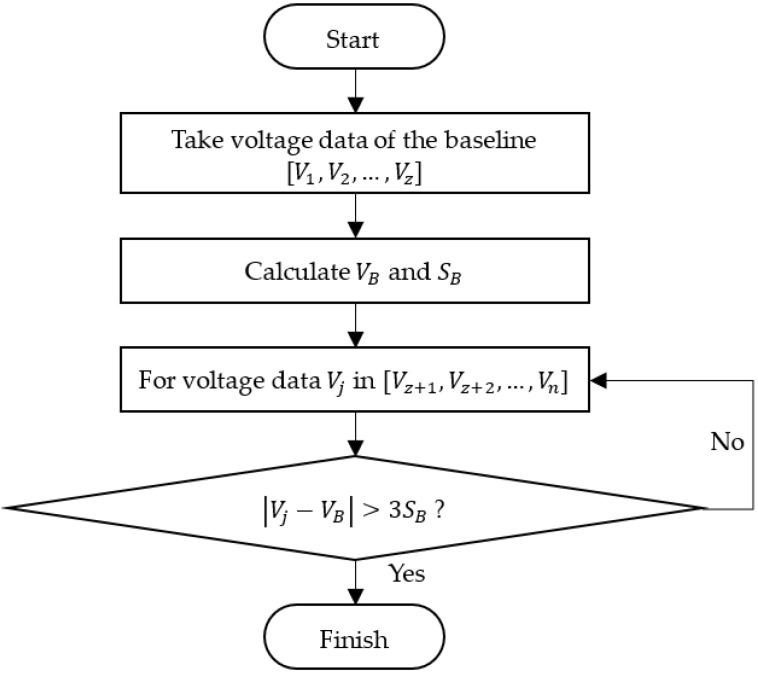
Flowchart of the “3σ” algorithm.

During the micromanipulation test, the voltage V(t) can be expressed as follows:

(12) $$V\left(t\right)=\frac{1}{s}F\left(t\right)+e\left(t\right),$$

where F(t) is the true value of the compression force, s is the sensitivity of the force transducer, and e(t) is a random noise. Before the onset of compression, F(t) is constant (zero), thus V(t) and e(t) have the same distribution during this period. Assuming the distribution is a normal distribution (the most common distribution [44] for noise), according to the three-sigma rule [45], the possibility (Pr) of V(t) falling away from the mean value (μ) of the baseline by more than three standard deviations (3σ) is at most 0.27%,

(13) Pr(|V(t)−μ|≥3σ)≤0.27%,

Thus, if the voltage value at a point starts to deviate from the baseline mean value by three standard deviations, it has a high possibility (99.73%) that the onset of compression begins; i.e., the first point when the voltage deviates from the baseline by three standard deviations can be located as the starting point. In practice, the μ and σ can be estimated by the average ($V_{B}$) and standard deviation ($S_{B}$) of the voltage data of the baseline. Then, a criterion is obtained to determine the starting point.

(14) $$\left|V_{m}-V_{B}\right|>3S_{B},$$

where m is the index of the starting point. The flowchart of the “3*σ*” algorithm is illustrated in Figure 4.

After initialization, the first *z* points of voltage $\left(V_{1},\;V_{2},\;\ldots ,\;V_{z}\right)$ are taken from the raw voltage data series $\left(V_{1},\;V_{2},\;\ldots ,\;V_{n}\right)$ as the baseline, from which the average $V_{B}$ and standard deviation $S_{B}$ are calculated using the following equation.

(15) $$V_{B}=\frac{1}{z}\sum_{i=1}^{z}V_{i},\;S_{B}=\sqrt{\frac{\sum_{i=1}^{z}\left(V_{i}-V_{B}\right)}{z-1}}$$

Then, the voltage data after *z*, $\left(V_{z+1},\;V_{z+2},\;\ldots ,\;V_{n}\right)$, are looked through for the first point when Inequation (14) is satisfied, whereafter the algorithm is stopped. The value of *z* can be estimated by the compression speed, sampling time, and the initial gap between the probe and the particle. Usually, *z* =20 is used, which is sufficiently accurate to determine $V_{B}$ and $S_{B}$.

#### 2.4.2. “3*σ* + Window” Algorithm

**Figure 5:**
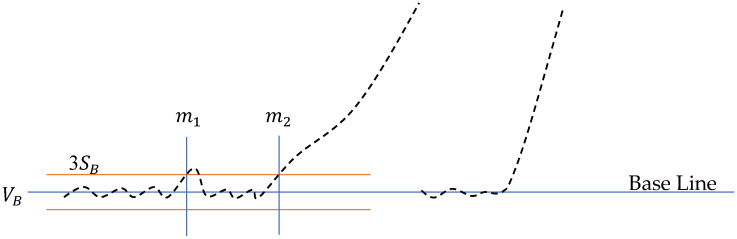
Problem of the “3*σ*” algorithm when impulse noise exists.

Normally, the “3*σ*” algorithm can locate the starting point successfully. However, if a pulse noise exists, the starting point may be determined incorrectly as illustrated in Figure 5. The point $m_{1}$ rather than $m_{2}$ will be misidentified as the starting point because of the impulse noise around $m_{1}$. Although smoothing the raw data by filtering can deal with the impulse noise, other key points such as the rupture value will be evened.

To tackle this problem, the “3*σ*” algorithm was modified by introducing a moving window with width w. A point m can be identified as the starting point only if all the points from it in the moving window fall away from $V_{B}$ by $3S_{B},$ which leads to the following criterion:

(16) $$\Lambda_{i=0}^{w-1}\left(\left|V_{m+i}-V_{B}\right|>3S_{B}\right)==true,$$

where Λ is the logical “and” Boolean operator. The width of the moving window w can be estimated as an integer corresponding to a percent of the diameter of the microparticle. As some brittle capsules and biological cells may rupture at a fractional deformation as small as 0.06 [8], a percent of 5% can ensure w less than the rupture deformation of most microparticles. Thus, w can be estimated using the following equation:

(17) $$w=\frac{0.05D}{vT_{s}},$$

#### 2.4.3. “3*σ* + Window + Hertz” Algorithm

The “3*σ* + window” algorithm can deal with most cases including those with random noise and impulse noise. However, it may underestimate the displacement when the voltage corresponding to three standard deviations of the baseline is just chosen as the starting point. This can result in a bigger value of the starting point index (m) so that the displacement will be underestimated, as it is related to the starting point by Equations (2), (7), and (8). The underestimation will be even worse when the signal to noise ratio is low. Following the same strategy described in [42], a mathematical model such as the Hertz model can be used to estimate the starting point (*m*) from the force-displacement data calculated using the “3*σ* + window” algorithm.

For diametrical compression of purely linear elastic microspheres, the Hertz model [1] relates the force to the displacement by the following equation:

(18) $$F=\frac{E\sqrt{D}}{3\left(1-\upsilon^{2}\right)}\delta^{\frac{3}{2}},$$

where E is the Young’s modulus, and υ is the Poisson’s ratio. Assume the force and displacement obtained from the “3*σ* + window” is $F^{\prime }$ and $\delta^{\prime }$, respectively, and the difference between the true displacement and the one obtained from the “3*σ* + window” is Δδ; then, Equation (18) can be written as

(19) $$F^{\prime }=k^{\prime }{\left(\delta^{\prime }+\Delta \delta \right)}^{\frac{3}{2}},$$

**Figure 6:**
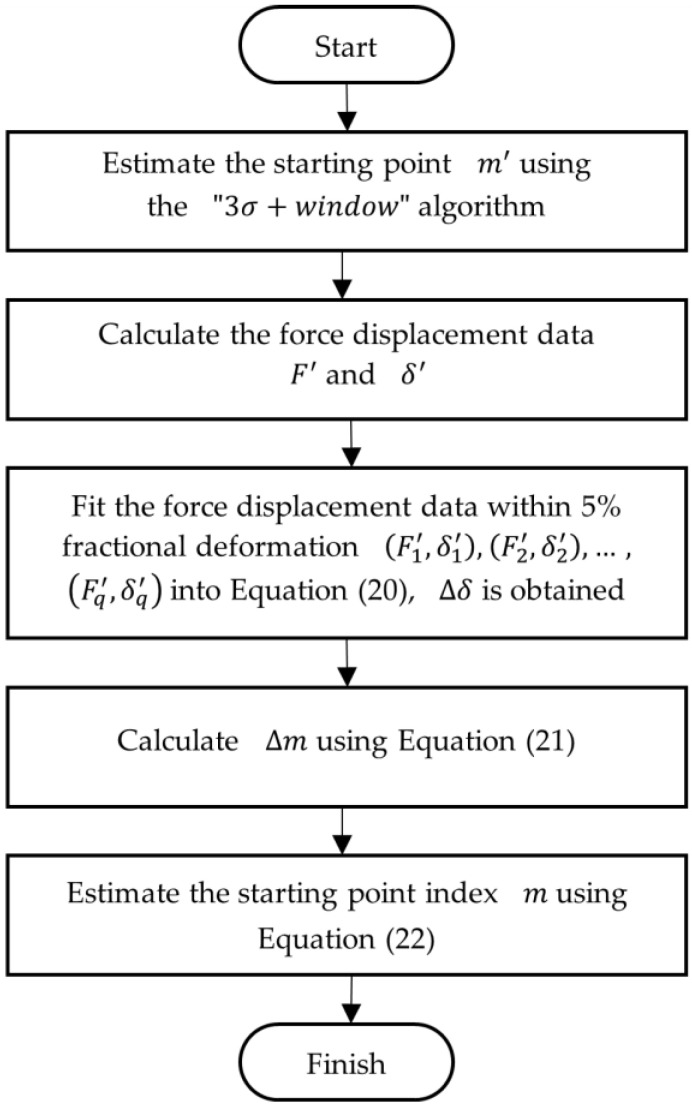
Flowchart of the “3*σ* + window + Hertz” algorithm.

Equation (19) can be transformed to

(20) $$\delta^{\prime }=k{\left(F^{\prime }\right)}^{\frac{2}{3}}-\Delta \delta ,$$

where $k=1/{\left(k^{\prime }\right)}^{2/3}$. Although the Hertz model is for purely linear elastic microspheres, it can be used to evaluate the true starting point by fitting into the initial compression data, such as within 5% deformation of the force-displacement data [23] obtained using the “3*σ* + window” algorithms. The flowchart of the algorithm can be illustrated by Figure 6. Firstly, a starting point index $m^{\prime }$ is estimated using the “3*σ* + window” algorithm, and the force-displacement data series $\left(\left(F_{1}^{\prime },\delta_{1}^{\prime }\right),\left(F_{2}^{\prime },\delta_{2}^{\prime }\right),\ldots ,\right)$] are calculated using Equations (7) and (8). Then, the force-displacement data within 5% deformation $\left(\left(F_{1}^{\prime },\delta_{1}^{\prime }\right),\;\left(F_{2}^{\prime },\delta_{2}^{\prime }\right),\ldots ,\;\left(F_{q}^{\prime },\delta_{q}^{\prime }\right)\right)$ are fit using Equation (20), and thus, Δδ is obtained, from which the different number Δm is estimated by Equation (21) to compensate the starting point.

(21) $$\Delta m=\frac{\left(CoD\right)\cdot \Delta \delta }{vT_{s}},$$

CoD is often explained as the proportion of the variance in the dependent variable that is predictable from the independent variable [46]. It also indicates the extent to which the dependent variable is predictable by the fitting model. In our case, the CoD represents how well the Hertz model can be used to present the relationship between the force and displacement data up to 5% fractional deformation. A value of 1.0 indicates a perfect fit, whilst a value of 0.0 would indicate that the Hertz model fails to model the data. Multiplying Δδ by CoD is expected only to use the predictable percent of Δδ to compensate the starting point. In other words, the compensated number Δm is not only calculated from the Δδ value estimated by the Hertz model but also from the “goodness” of the fit, i.e., how well the Hertz model can fit the data. In this way, Equation (21) adjusts the compensation extent automatically according to the goodness of fit (CoD), which makes the compensation algorithm intelligent.

Finally, the index of the starting point can be obtained by Equation (22).

(22) $$m=m^{\prime }-\Delta m,$$

### 2.5. Algorithms to Locate the Rupture Point

#### Maximum-Deceleration Algorithm

Normally, the voltage drops most dramatically just after the rupture point so that it can be identified by looking for the maximum deceleration through the voltage series. Practically, the deceleration is calculated from the following equation:

(23) $$\Delta V_{i}=\frac{V_{i+1}+V_{i+2}}{2}-V_{i},\;i=1,2,\cdots ,n-2,$$

where $\left(V_{i+1}+V_{i+2}\right)/2$ rather than $V_{i+1}$ is used to filter the data slightly to reduce the possible impact of random noise.

**Figure 7:**
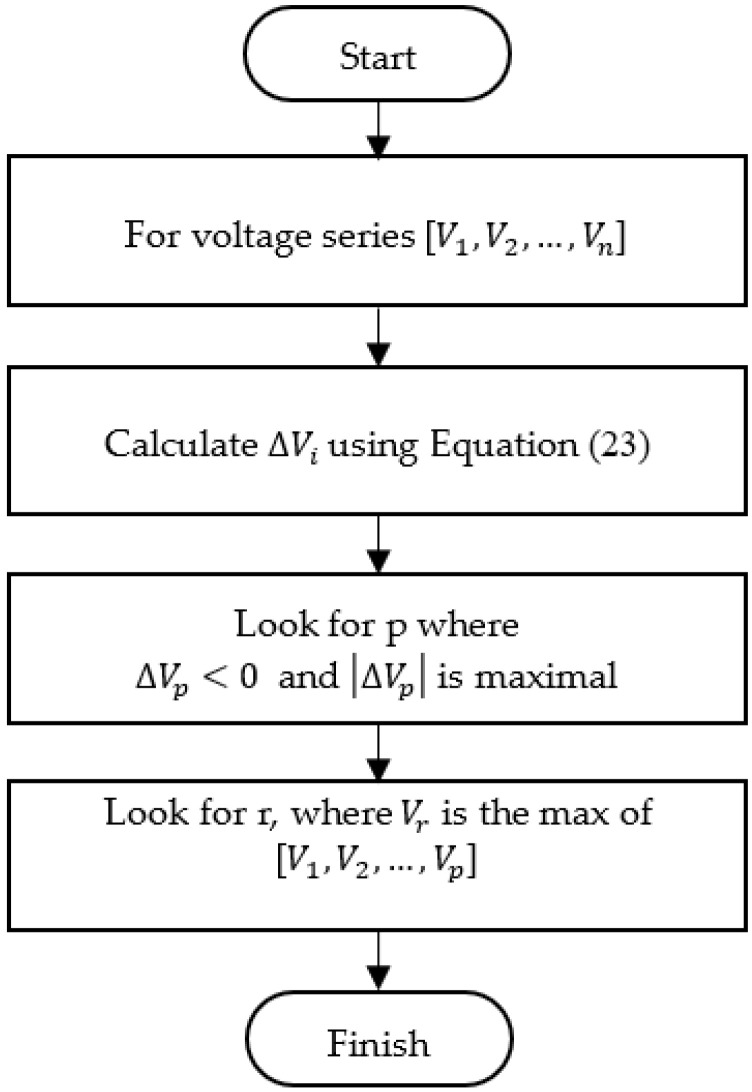
Flowchart of the maximum-deceleration algorithm.

The flowchart of the algorithm is illustrated in Figure 7. Initially, the drop (deceleration) series is calculated from the voltage series. Then, the point with the maximum drop (point p) is found, and the rupture point (r) is located as the peak point before p.

### 2.6. Development of the Software Package

Visual Studio 2017 Community and .NET from Microsoft were chosen as the main development platform to develop the automatic data analysis software package. The user interface (UI) module, report-generating module, and main program module are mainly developed with the C# language, and the data read and conversion module, data processing and analysis module are mainly developed with the F# language. Besides, some open-source software libraries, such as Math.Net and EEPlus, are used to facilitate the software development. The open-source libraries used are listed in Table 1.

## 3. Results and Discussion

### 3.1. Performance of the Algorithms

**Figure 8:**
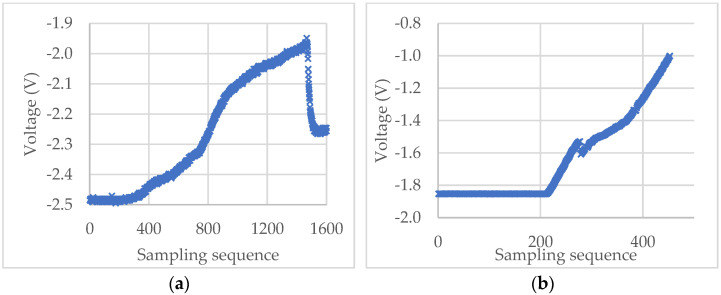
Experimental voltage-sampling sequence curves of a self-sensing microcapsule (**a**) and a porous PS microsphere (**b**).

**Figure 9:**
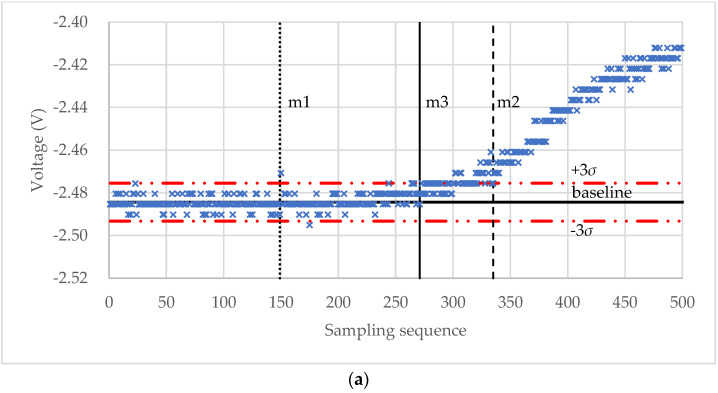

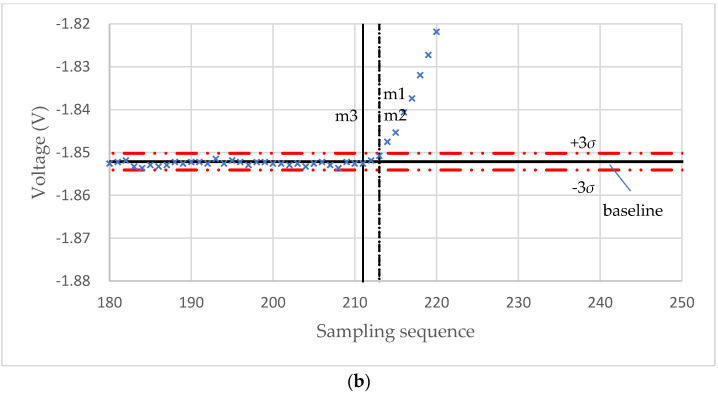
Starting point values found by the three algorithms. (**a**) Results for a self-sensing microcapsule and (**b**) results for a porous PS microsphere.

For the experimental raw voltage data of a microcapsule for self-sensing shown in Figure 8a, the starting point m1, m2, and m3 found by the “3*σ*”, “3*σ* + window”, and “3*σ* + window + Hertz” algorithms, respectively, are shown in Figure 9a. The diameter of the microcapsule was 87.6 μm. It can be seen that for this set of experimental data, the result of the “3*σ*” algorithm seems to underestimate the starting point because of the impulse noise, whilst the result of the “3*σ* + window” appears to overestimate the starting point. The starting point found by the “3*σ* + window + Hertz” algorithm looks more reasonable as the voltage starts to increase around this point. However, when no impulse noise exists, the starting point values obtained from the “3*σ*” (m1) and “3*σ* + window” (m2) algorithms are the same. For instance, for the raw voltage data of a porous PS microsphere in Figure 8b, the starting point is m1 = m2 = 213 as shown in Figure 9b. In both cases, the rupture points are successfully identified by the maximum-deceleration algorithm.

**Figure 10:**
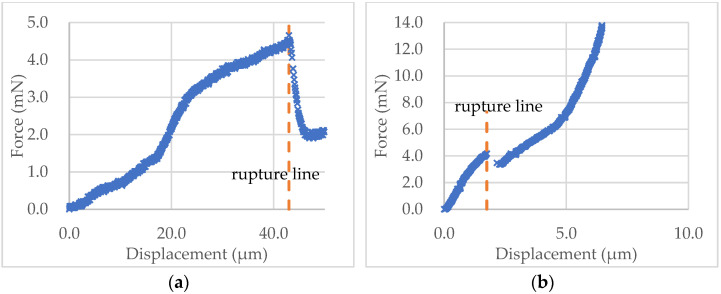
The force-displacement curves obtained using the starting point of m3 and the rupture point determined automatically for the experimental data of the self-sensing microcapsule (**a**) and the PS microsphere (**b**) in Figure 8a,b, respectively.

In the following analysis, the staring point (m3) was found by the “3*σ* + window + Hertz” algorithm, as it is more reasonable as discussed above. The force-displacement data of the microcapsule in Figure 8a and microsphere in Figure 8b were calculated using Equations (7) and (8), and their curves are shown in Figure 10a,b, respectively. It can be seen from Figure 10a that the force-displacement curve of the self-sensing microcapsule is not very smooth, with some local peaks before rupture that might be due to the roughness of the out-layer PUF [5,47]. The out layer could crack several times before the rupture point shown in Figure 10a, where the inner shell was ruptured, and the force dropped sharply. In contrast, the force-displacement curve of the PS microsphere is quite smooth before the rupture, as its shell was smooth [43]. However, the force at the rupture point did not drop as dramatically as the self-sensing microcapsule since there was no release of any mateiral from the PS microsphere at the rupture point.

**Figure 11:**
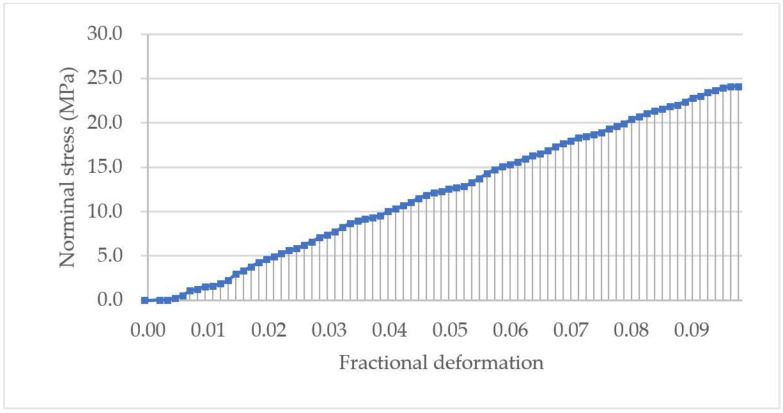
Nominal stress versus fractional deformation up to rupture of the PS microsphere in Figure 8b. The toughness corresponds to the area under the curve, i.e., the integration of the nominal rupture stress over the fractional deformation using Equation (11). The starting point M was found using the “3*σ* + window + Hertz” algorithm (m3 in Figure 8b).

The nominal stress-fractional deformation data up to rupture of the PS microsphere in Figure 8b was calculated using Equation (9) and (10), and its curve is shown in Figure 11. The starting point was m3 in Figure 9b, found using the “3*σ* + window + Hertz” algorithm. The toughness of the particle was 1.15 MPa, calculated using the trapezoidal numerical integration in Equation (11), corresponding to the area under the curve up to rupture.

The experimental data of 50 self-sensing microcapsules and 50 PS microspheres were analysed utilizing the developed software package, and a manual analysis was also carried out for comparison. The average and standard error of the calculated rupture strength parameters for the two samples are shown in Table 2 and Table 3. It appears that for the two samples, the average rupture force values from the automatic data analyses are all the same as those from the manual analysis, which shows that the “maximum-deceleration” algorithm is very robust to locate the rupture point. So are the average values of nominal rupture stress, nominal rupture tension and the toughness as the former two parameters are calculated from the rupture force and the diameter of the microparticle. Although the toughness is related to the fractional deformation, which depends on the starting point, the force changes little around the starting point, so the effect of the initial integration of the nominal stress over the fractional deformation on the toughness value is negligible. Thus, the average values of the toughness from the four analyses show the same results. The values of the displacement at rupture from “3*σ* + window” and “3*σ* + window + Hertz” overlap with the results from the manual analysis. Because of the appearance of impulse noises, the values of displacement at rupture and deformation at rupture from “3*σ*” algorithm appear to be different significantly from the manual analysis results. It was found that the starting points for nearly half (24/50) of the tested self-sensing microcapsules and 17/50 of the porous PS microspheres were not correctly identified using the “3*σ*” algorithm.

Based on the data of these two samples, the results obtained from using “3*σ* + window” and “3*σ* + window + Hertz” algorithms have no significant difference from the manual results so that they both can be used in the automatic analysis of the rupture strength of microparticles.

### 3.2. Further Discussion

From the mean values in Table 2 and Table 3, it appears that the fractional deformation at rupture of the self-sensing microcapsules is quite big (nearly 50%) in comparison with that of the porous PS microspheres (just around 12%). This indicates that the self-sensing microcapsules with double PUF-PU shells showed a ductile failure behaviour, while the porous PS microspheres showed a brittle failure behaviour [8]. However, the nominal rupture stress of the former (0.85 MPa) is much smaller than the latter (26.4 MPa). It is the same with the toughness, as it is related to the nominal stress versus fractional deformation up to rupture. This may result from the large difference in the particle sizes between the two samples since the nominal rupture stress normally decreases with the increasing particle diameter [48]. The values of the diameter for the self-sensing microcapsules and PS microspheres are 86.2 ± 3.1 μm and 11.1 ± 0.4 μm, respectively.

Moreover, the nominal rupture tension of the self-sensing microcapsules (53.9 μN/μm) is also much smaller than that of the porous PS microspheres (227.9 μN/μm). This is reasonable, as the former had a liquid core surrounded by a solid shell with thickness between 200 and 500 nm [5], whilst the latter were solid with a few pores on the surface [43].

**Figure 12:**
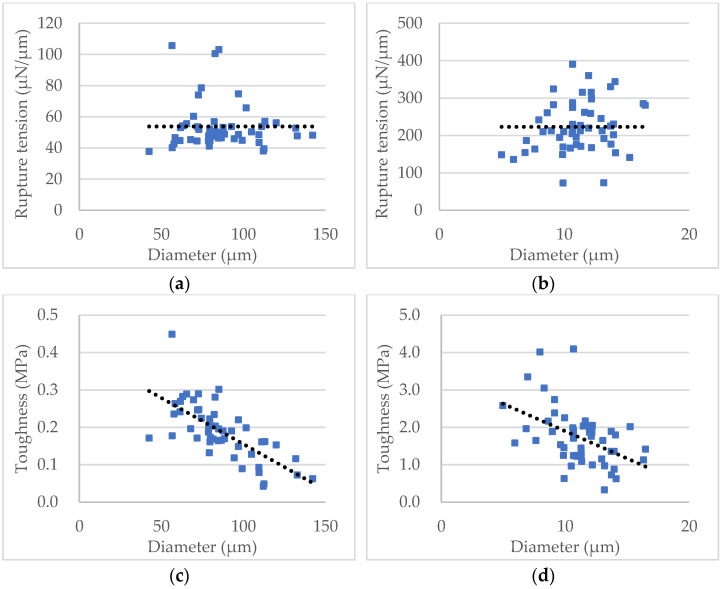
Data of the nominal rupture tension and toughness versus diameter of the two samples. (**a**) Nominal rupture tension of the self-sensing microcapsules. (**b**) Nominal rupture tension of the porous PS microspheres. (**c**) Toughness of the self-sensing microcapsules. (**d**) Toughness of the porous PS microspheres. Each fitted line (dotted) only indicates the trend, with 95% confidence.

The nominal rupture tension and the toughness versus diameter of the two samples of individual microspheres are illustrated in Figure 12. Statistical analysis of the data shows that the nominal rupture tension does not change with diameter for each sample significantly (Figure 12a,b), which can be used to compare the mechanical strength between samples with particles of different sizes. In contrast, the toughness decreases with the diameter, which indicates bigger particles were weaker than smaller ones (Figure 12c,d), similar to the nominal rupture stress [48].

### 3.3. Comparison with Other Algorithms

The standard deviation was used in several algorithms to evaluate the noise level of the raw data and to help estimate the parameters of the algorithms to identify CP for AFM force data [38,41,42,49]. A moving window was also introduced to help the identification of the CP [41,42]. However, it was only used for local regression rather than dealing with the impulse noise as addressed by the “3*σ*+ window” algorithm. Besides, the width of the moving window needs to be set manually in the reported algorithms, whilst it is estimated automatically by Equation (17) in the “3*σ* + window” and “3*σ* + window + Hertz” algorithms developed in this work. Furthermore, the algorithm in [42] pre-estimates a CP* with a threshold of five standard deviations of the baseline values and then determines the CP by fitting force-displacement data into a Hertz-like model from CP* to an indentation depth empirically determined by the stiffness of the force curve. The “3*σ* + window + Hertz” algorithm also pre-estimates a prone starting point $m^{\prime }$ followed by the regression of the force-displacement data within 5% fractional deformation to the Hertz model to determine the real starting point m. However, these two algorithms have two main differences. One is that the “3*σ* + window” algorithm is used to estimate the prone starting point, which can well deal with the impulse noises in the “3σ + window + Hertz” algorithm, whereas CP* is just estimated with a threshold of five standard deviations of the baseline values [42] that may result in a wrong value when the impulse noise greater than the threshold exists before the real CP. The other difference is that after the Hertz regression, the CoD is used in Equation (21) to adjust the degree of the compensation automatically so that when the tested material is not linear ealstic, fewer points will be compensated to $m^{\prime }$. In contrast, the algorithm reported in [42] was designed for linear elastic materials and cannot adjust automatically for other mechanical behaviours of the tested materials. Besides, using the “maximum-deceleration” algorithm for the detection of rupture point in this work requires no parameter to be adjusted and is fully automatic, which is advantageous.

## 4. Conclusions

In this study, a data analysis software package was developed to analyse the rupture strength of microparticles automatically from the experimental data of micromanipulation measurements. Three algorithms were developed to find the starting point of the compression data, i.e., the “3*σ*”, “3*σ* + window” and “3*σ* + window + Hertz”. The “3*σ*” algorithm determines the starting point where the voltage of a point deviates from the mean of baseline ($V_{B}$) by three standard deviations ($3S_{B}$), whilst in the “3*σ* + window” algorithm, a point is determined as the starting point only if the following w points (including this point) all deviate from $V_{B}$ by $3S_{B}$. In the “3*σ* + window + Hertz” algorithm, the starting point is further adjusted by fitting the force-displacement data corresponding to very small deformations (up to 5% fractional deformation) into the Hertz model to compensate the underestimation of the displacement corresponding to the three standard deviations. One algorithm based on the maximum deceleration of the voltage series was developed to determine the rupture point. The results show that the combination of the “3*σ* + window” or “3*σ* + window + Hertz” algorithm with the “maximum-deceleration” algorithm can produce results that are in excellent agreement with those obtained manually, and there is no significant difference between them. Moreover, all the developed algorithms work fully automatically without any parameter modification.

For analysing 50 microparticles in a typical sample, the time spent on analysing the rupture strength parameters manually was from 2 to 3 h. In contrast, it took less than 20 min to analyse the same data automatically using the software package developed in this work. It is believed that this software package can also be used to analyse the force-displacement data obtained using conventional mechanical testing machines for macro-scale materials, which can have a wide range of applications.

## Figures and Tables

**Table 1:** Open-source libraries used in the development of the software package.

Library	Version	License
Daria	2.0.2	MIT
EEPlus	4.5.3.2	LGPL-3.0-or-later
ExcelDataReader	3.6.0	MIT
ExcelDataReader.DataSet	3.6.0	MIT
Math.NET Numerics	4.8.0	https://numerics.mathdotnet.com/License.html (accessed on 8 May 2022).
Accord.NET	3.8.0	http://accord-framework.net/license.txt (accessed on 8 May 2022).

**Table 2:** Rupture strength of the self-sensing microcapsules obtained from different algorithms.

Algorithm	Diameter (μm)	Displacement at Rupture (μm)	Rupture Force (mN)	Deformation at Rupture (%)	Nominal Rupture Stress (MPa)	Nominal Rupture Tension (μN/μm)	Toughness (MPa)
Manual	86.2 ± 3.1	40.5 ± 1.4	4.61 ± 0.22	47.7 ± 1.1	0.85 ± 0.05	53.8 ± 2.2	0.19 ± 0.01
3*σ*	86.2 ± 3.1	44.7 ± 1.7	4.61 ± 0.22	52.6 ± 1.5	0.85 ± 0.05	53.8 ± 2.2	0.19 ± 0.01
3*σ* + Window	86.2 ± 3.1	39.0 ± 1.4	4.61 ± 0.22	45.7 ± 1.1	0.85 ± 0.05	53.8 ± 2.2	0.19 ± 0.01
3*σ* + Window + Hertz	86.2 ± 3.1	41.0 ± 1.4	4.61 ± 0.22	48.2 ± 1.1	0.85 ± 0.05	53.8 ± 2.2	0.19 ± 0.01

**Table 3:** Rupture strength of the porous PS microspheres obtained from different algorithms.

Algorithm	Diameter (μm)	Displacement at Rupture (μm)	Rupture Force (mN)	Deformation at Rupture (%)	Nominal Rupture Stress (MPa)	Nominal Rupture Tension (μN/μm)	Toughness (MPa)
Manual	11.1 ± 0.4	1.3 ± 0.1	2.53 ± 0.15	12.0 ± 0.4	26.4 ± 1.2	223.0 ± 9.9	1.73 ± 0.11
3*σ*	11.1 ± 0.4	2.5 ± 0.3	2.53 ± 0.15	21.9 ± 2.3	26.4 ± 1.2	223.0 ± 9.9	1.73 ± 0.11
3*σ* + Window	11.1 ± 0.4	1.3 ± 0.1	2.53 ± 0.15	11.9 ± 0.4	26.4 ± 1.2	223.0 ± 9.9	1.73 ± 0.11
3*σ* + Window + Hertz	11.1 ± 0.4	1.4 ± 0.1	2.53 ± 0.15	12.7 ± 0.4	26.4 ± 1.2	223.0 ± 9.9	1.73 ± 0.11

## Data Availability

Not applicable.

## Nomenclature

c	Compliance of the force transducer (${\mathrm{mN}}^{-1}$)
CoD	Coefficient of determination
D	Initial diameter of the single microparticle (m)
e(t)	Random noise
E	Young’s modulus (Pa)
$F,\;F_{i}$, F(t)	Compression force (N)
$F_{r}$	Rupture force (N)
$F^{\prime }$	Estimated force in the “3*σ* + window + Hertz” algorithm (N)
$(\left(F_{1}^{\prime },\delta_{1}^{\prime }\right),\;\left(F_{2}^{\prime },\delta_{2}^{\prime }\right),\;\ldots ,\;\left(F_{p}^{\prime },\delta_{p}^{\prime }\right)$)	Estimated force-displacement series in the “3*σ* + window + Hertz” algorithm
k, $k^{\prime }$	$k=1/{\left(k^{\prime }\right)}^{2/3}$, used in the “3*σ* + window + Hertz” algorithm
m	Starting point index
$m_{1}$	Starting point index found by the “3*σ*” algorithm
$m_{2}$	Starting point index found by the “3*σ* + window” algorithm
$m_{3}$	Starting point index found by the “3*σ* + window + Hertz” algorithm
$m^{\prime }$	Estimated starting point index in the “3*σ* + window + Hertz” algorithm
Δm	Values to compensate starting point index in the “3*σ* + window + Hertz” algorithm
Pr	Possibility
r	Rupture point index
s	Sensitivity of the force transducer (${\mathrm{NV}}^{-1}$)
$S_{B}$	Standard deviation of baseline voltage (V)
$T_{C}$	Particle toughness (Pa)
$T_{r}$	Nominal rupture tension (${\mathrm{Nm}}^{-1}$)
$T_{s}$	Sampling time (s)
v	Compression speed (m$s^{-1}$)
V, $V_{i}$, $V_{m}$, V(t)	Voltage (V)
$\left(V_{1},\;V_{2},\;\ldots ,\;V_{z}\right)$	Voltage series of the baseline (V)
$\left(V_{1},\;V_{2},\;\ldots ,\;V_{n}\right)$	Raw voltage data series (V)
$V_{B}$	Average voltage of the baseline (V)
$V_{r}$	Voltage corresponding to rupture (V)
ΔV, $\Delta V_{i}$	Voltage deceleration in the “maximum-deceleration” algorithm (V)
w	Width of moving window in the “3*σ* + window” algorithm
*z*	Number of initial voltage points to estimate baseline values
***Greek letters***	
$\delta ,\;\delta_{i}$	Displacement (m)
$\delta_{r}$	Displacement at rupture (m)
$\delta^{\prime }$	Estimated displacement in the “3*σ* + window + Hertz” algorithm (m)
Δδ	Difference between the true displacement and the one obtained from the “3*σ* + window” algorithm (m)
ε, $\varepsilon_{i}$	Fractional deformation
$\varepsilon_{r}$	Fractional deformation at rupture
μ	Mean value
υ	Poisson’s ration
3σ	Three sigma, three standard deviations
σ, $\sigma_{i}$	Nominal stress (Pa)
$\sigma_{r}$	Nominal rupture stress (Pa)
